# Preclinical Evaluation of the Novel BTK Inhibitor Acalabrutinib in Canine Models of B-Cell Non-Hodgkin Lymphoma

**DOI:** 10.1371/journal.pone.0159607

**Published:** 2016-07-19

**Authors:** Bonnie K. Harrington, Heather L. Gardner, Raquel Izumi, Ahmed Hamdy, Wayne Rothbaum, Kevin R. Coombes, Todd Covey, Allard Kaptein, Michael Gulrajani, Bart Van Lith, Cecile Krejsa, Christopher C. Coss, Duncan S. Russell, Xiaoli Zhang, Bridget K. Urie, Cheryl A. London, John C. Byrd, Amy J. Johnson, William C. Kisseberth

**Affiliations:** 1 Department of Veterinary Biosciences, College of Veterinary Medicine, The Ohio State University, Columbus, Ohio, United States of America; 2 Department of Veterinary Clinical Sciences, College of Veterinary Medicine, The Ohio State University, Columbus, Ohio, United States of America; 3 Acerta Pharma BV, Oss, Netherlands; 4 Department of Biomedical Informatics, College of Medicine, The Ohio State University, Columbus, Ohio, United States of America; 5 Division of Pharmaceutics and Pharmaceutical Chemistry, College of Pharmacy, The Ohio State University, Columbus, Ohio, United States of America; 6 Department of Biomedical Sciences, College of Veterinary Medicine, Oregon State University, Corvallis, Oregon, United States of America; 7 Center for Biostatistics, The Ohio State University, Columbus, Ohio, United States of America; 8 Pittsburgh Veterinary Specialty and Emergency Center, Pittsburgh, Pennsylvania, United States of America; 9 Division of Hematology, Department of Internal Medicine, College of Medicine, The Ohio State University, Columbus, Ohio, United States of America; Cornell University, UNITED STATES

## Abstract

Acalabrutinib (ACP-196) is a second-generation inhibitor of Bruton agammaglobulinemia tyrosine kinase (BTK) with increased target selectivity and potency compared to ibrutinib. In this study, we evaluated acalabrutinib in spontaneously occurring canine lymphoma, a model of B-cell malignancy similar to human diffuse large B-cell lymphoma (DLBCL). First, we demonstrated that acalabrutinib potently inhibited BTK activity and downstream effectors in CLBL1, a canine B-cell lymphoma cell line, and primary canine lymphoma cells. Acalabrutinib also inhibited proliferation in CLBL1 cells. Twenty dogs were enrolled in the clinical trial and treated with acalabrutinib at dosages of 2.5 to 20mg/kg every 12 or 24 hours. Acalabrutinib was generally well tolerated, with adverse events consisting primarily of grade 1 or 2 anorexia, weight loss, vomiting, diarrhea and lethargy. Overall response rate (ORR) was 25% (5/20) with a median progression free survival (PFS) of 22.5 days. Clinical benefit was observed in 30% (6/20) of dogs. These findings suggest that acalabrutinib is safe and exhibits activity in canine B-cell lymphoma patients and support the use of canine lymphoma as a relevant model for human non-Hodgkin lymphoma (NHL).

## Introduction

B-cell receptor (BCR) signaling is a critical factor in the progression of many subtypes of B-cell NHL. This signaling is driven through a variety of mechanisms, including BCR binding to self or foreign antigen[1–7], overexpression or aberrant expression of signal transducers[8,9], and oncogenic somatic mutations driving distal signaling pathways[10,11]. Regardless of the mechanism of activation, signaling via the BCR and the key proximal signaling molecule BTK leads to increased cell proliferation, survival, and homing to the microenvironment[12–14]. Several targeted therapeutics that inhibit this signaling pathway are in development, including those that target BTK. The clinical activity of IMBRUVICA^®^ (ibrutinib), a first-in-class BTK inhibitor, has validated BTK as a therapeutic target in B-cell malignancies.

Second-generation BTK inhibitors with more selective kinase activity profiles are being developed, including acalabrutinib (Acerta Pharma BV, Oss, the Netherlands). Acalabrutinib covalently binds BTK at the cysteine-481 residue and inhibits with greater *in vivo* potency and selectivity than ibrutinib [15] and also has demonstrated efficacy in early clinical trials involving relapsed and refractory CLL [16]. Preclinical development of ibrutinib included treatment of dogs with B-cell lymphoma [17], perhaps because many similarities to human NHL are recapitulated in canine B-cell lymphoma, including histologic characteristics and response to chemotherapeutics. The life expectancy in untreated dogs with aggressive disease is ~6 weeks [18]. In humans, DLBCL is the most common subtype of NHL, and the advent of genomic technologies has allowed molecular subtyping of this heterogeneous disease process in both people and dogs [19–21]. Gene expression profiling (GEP) of canine DLBCL demonstrates that it can be genetically subcategorized, similar to its human counterpart [21], and that canine DLBCL can be separated into germinal center B-cell (GCB)-like and activated B-cell (ABC)-like subgroups [20]. Similar to DLBCL in humans, differences in progression-free and overall survival were found between the ABC-like and GCB-like canine patients.

For these reasons, we elected a canine model of B-cell NHL to evaluate the pharmacodynamic effects of acalabrutinib *in vitro* and *in vivo*. In this study, we explore BCR signaling activity in canine neoplastic B-cells, and demonstrate that acalabrutinib inhibits BTK signaling in vitro, resulting in cytotoxic and anti-proliferative effects similar to those reported with ibrutinib [22]. We compare the GEP in the canine lymphoma cell line CLBL1, with GEP from primary canine B-cell lymphomas, to establish ABC-like or GCB-like subtype in the in vitro model. Finally, we report results of a phase I clinical trial of acalabrutinib in canine B-cell lymphoma, showing clinical benefit in a significant proportion of patients. Overall, our data demonstrate that acalabrutinib is a clinically well-tolerated and effective BTK inhibitor in dogs.

## Materials and Methods

### Reagents

Acalabrutinib was provided by Acerta Pharma (Oss, Netherlands). Fluorescein isothiocyanate (FITC)-labeled annexin-V and propidium iodide (PI) for flow cytometry were purchased from BD Pharmingen (San Diego, CA).

### Patient sample processing and cell culture

Biopsies and fine needle aspirates (FNAs) were collected from affected peripheral lymph nodes of canine lymphoma patients. The canine B-cell lymphoma line CLBL1 was a generous gift from Barbara Rütgen (Vienna, Austria) [23]. Erythrocyte contamination was removed from FNAs by ammonium chloride lysis. Peripheral blood samples were processed by density gradient centrifugation, followed by positive magnetic enrichment of B-cells using anti-canine CD21-PE antibody (clone CA2.1D6, Bio-Rad, Hercules, CA), and anti-phycoerythrin microbeads (Miltenyi Biotec, San Diego, CA). Enrichment was confirmed by flow cytometry. All samples were snap-frozen and stored in liquid nitrogen. Cell lines and primary lymphoma cells were incubated at 37°C with 5% CO_2_ in RPMI-1640 medium enriched with 10% fetal bovine serum (Sigma-Aldrich, St. Louis, MO), 100 U/mL penicillin/100 μg/mL streptomycin (Sigma-Aldrich), and 2 mM L-glutamate (Invitrogen, Carlsbad, CA). For *in vitro* signaling, apoptosis and proliferation experiments, cells were incubated with acalabrutinib for 1 hour followed by 2 washes with phosphate buffered saline (PBS). For 120 hour experiments, cells were treated every 24 hours, washed, and returned to the culture plate.

### Immunoblot analysis

Cell lines and primary cells were treated with acalabrutinib and stimulated with plate-bound anti-human IgM (MP Biomedicals; Santa Ana, CA). Plates were prepared by incubating a 10 μg/mL IgM solution in PBS for 6 to 12 hours at 4°C, and then rinsing with PBS. Whole cell lysates were prepared as previously described [24], followed by polyacrylamide gel electrophoresis and transfer of proteins to nitrocellulose membranes. The following polyclonal antibodies were used to detect protein on immunoblots: anti-phospho-PLCG2 (Tyr 1217, Cat. #3871), anti-PLCG2 (Cat. #3872), anti-phospho-IKBA (Ser32, Cat. #2859), anti-IKBA (Cat. #4812), anti-phospho-ERK1/2 (Thr202/Tyr204, Cat. #9101), anti-ERK1/2 (Cat. #9102), anti-phospho-AKT (Thr308, Cat. #9257), and anti-AKT (Cat. #9272), anti-phospho-NFKB P65 (Ser536, Cat. #3031), anti-NFKB P65 (Cat. #3034)(Cell Signaling Technologies; Danvers, MA), anti-phospho-BTK (Tyr223, Cat. #ab68217, Abcam, Cambridge, MA), and anti-BTK (cat. #B3187, Sigma-Aldrich).

### Viability and proliferation assays

Cell viability was measured using annexin-V/PI flow cytometry (Beckman-Coulter; Miami, FL). Cell proliferation was measured using Click-iT® Plus EdU Alexa Fluor® 647 Flow Cytometry Assay Kit (Life Technologies, Grand Island, NY) according to manufacturer instructions. Staining and analysis were performed as previously described by our laboratory [25].

### RNA extraction and gene expression profiling

Total RNA was isolated using the Trizol method and DNase treated. RNA integrity was interrogated using the Agilent 2100 Bioanalyzer (Agilent Technologies, Palo Alto, CA). A 2 μg aliquot of total RNA was linearly amplified and labeled using the BioArray High Yield RNA Transcript labeling kit (Enzo Life Sciences). Then, 15 μg of labeled cRNA was fragmented following the manufacturer instructions. Labeled cRNA targets were hybridized to Affymetrix GeneChip® Canine Genome 2.0 array for 16 hours at 45°C rotating at 60 rpm. Arrays were washed and stained using the Fluidics Station 450 and scanned using the GeneChip Scanner 3000 (Affymetrix). For gene expression analysis, arrays were normalized. The microarray data have been deposited in the NCBI Gene Expression Omnibus (GSE81110).

For clustering, we downloaded supplementary microarray data (GSE43664) from GEO, then merged the microarray data for CLBL1 and processed the combined data using the robust multiarray averaging (RMA) method in version 3.1.2 of the R statistical programming environment. We then applied the median polish algorithm to the subset of 1180 canine probe sets found to separate two subtypes of canine B-cell lymphoma [20]. Samples were clustered using centroid linkage and a distance metric based on un-centered correlation. To confirm the cluster assignment, we computed distance from CLBL1 to the centers of both the GCB and ABC clusters using three methods: Euclidean, Pearson correlation, and un-centered correlation.

### Study design

This clinical trial was a multicenter open-label, nonrandomized, sequential group, dose-escalation study of acalabrutinib in companion dogs with spontaneous B-cell lymphoma. The study was approved by The Ohio State University Veterinary Medical Center Clinical Research Advisory Committee and Institutional Animal Care and Use Committee. Similar permission was obtained at Pittsburgh Veterinary Specialty and Emergency Center. Owners gave written informed consent prior to patient enrollment. Treatment-naïve dogs and dogs with prior therapies and a confirmed diagnosis of new or relapsed B-cell lymphoma (stage ≥ 2) were eligible. Diagnosis of B-cell lymphoma was based on lymph node fine needle aspirate (FNA) cytology and/or histologic evaluation of lymph node biopsies and flow cytometry, immunophenotyping, immunohistochemistry, or PCR for antigen receptor rearrangement [26]. Dogs met all eligibility criteria, including: ≥1 year of age; performance status of 0–1; adequate organ function as determined by routine bloodwork; ≥2 weeks from previous anti-neoplastic treatments (chemotherapy, radiation therapy, surgery, or other investigational therapies) and complete resolution of toxicities from prior treatments. Dogs with T-cell lymphoma were excluded. Prior to enrollment, dogs underwent screening tests including complete blood count, serum biochemistry profile, urinalysis, thoracic radiographs, tumor immunophenotyping and lymph node biopsy for evaluation of histomorphologic subtype.

Dogs with spontaneous B-cell lymphoma that failed conventional therapy or for which there was no therapeutic alternative, or for which conventional therapy was not desired by the owner were enrolled in the study. Dogs were administered acalabrutinib orally (PO) once or twice daily for a 7 day cycle and continued until clinical progression. Assessment of patients for tumor response was performed weekly by direct tumor measurement, or through the use of imaging techniques such as radiography or ultrasound. Assessment of clinical toxicities and tumor response were performed at each visit. Dogs were evaluated for hematologic and biochemical toxicities every 7 days with routine bloodwork (CBC, serum biochemical profile). The initial dose of 2.5 mg/kg orally once daily was based on a previous data from a toxicology study in healthy purpose bred dogs (data not shown) and dose escalations were set at 5, 10, 15, and 20 mg/kg once or twice daily in cohorts of 6 dogs until either full BTK occupancy was achieved, dose limiting toxicity (DLT) was identified, or if objective tumor responses were noted in greater than 50% of a particular cohort. The DLT was considered to be any grade 3 or 4 hematologic or non-hematologic toxicity based on established VCOG-CTCAE v1.1 criteria [27]. Disease progression or clinical signs definitely related to disease were not considered adverse events (AEs). The maximum tolerated dose (MTD) was considered to be one dose below that at which DLT occurred. Intrapatient dose escalation was permitted in dogs with a modified ECOG performance score of 0–1 in the face of disease progression or after day 14 pharmacokinetic assessment had been completed [28,29]. Plasma samples were obtained for pharmacokinetic analysis and peripheral blood mononuclear cells (PBMCs) and lymph node FNAs were obtained for pharmacodynamic analysis.

### Concomitant medications on clinical trial subjects

Supportive care was provided as needed to dogs while enrolled in this trial. Permitted concomitant medications included famotidine, omeprazole, maropitant and/or metronidazole. Prednisone was administered at a maximum dose of 0.5 mg/kg daily to treat tumor-related inappetence. Dogs on prednisone at enrollment were permitted to continue treatment if disease progression had occurred while on prednisone. Prednisone use was allowed during the course of the study in dogs with inappetence, regardless of prior prednisone use. Additional supportive care was administered as clinically indicated. Intravenous pentobarbital injection was used for euthanasia, as elected by owner.

### Pharmacokinetic analysis

Plasma samples were collected before dosing on days 0 and 14, and at 0.5, 1, 2, 4, 6, 8, 12 and 24 hours after dose administration on day 14. One hundred microliters of heparinized blood was mixed with 600 μL of acetonitrile and stored at -80°C within 20 minutes of collection. Plasma samples were analyzed using a qualified HPLC-MS/MS method for acalabrutinib. Plasma concentration-time data was analyzed by non-compartmental methods using default settings and the NCA analysis object in Phoenix WinNonlin v6.3 (Pharsight, Mountain View, CA). C_max_/Dose and T_max_ were determined following visual inspection of the plasma concentration versus time plots of individual patient data.

### BTK occupancy analysis

Target occupancy analysis was performed on peripheral blood and fine needle aspirates from lymph nodes. Ninety-six-well Optiplate (Perkin Elmer, Waltham, MA) plates were coated with 125 ng/well anti-BTK Ab (BD Biosciences, San Jose, CA) and blocked with BSA (Sigma-Aldrich). A biotinylated analogue of acalabrutinib was added to cell lysates to bind unoccupied kinase sites on immobilized BTK. Cell lysates were then plated in duplicate and incubated in presence or absence of 1 μM acalabrutinib to identify minimal and maximal signals. Binding was detected by incubation with 10 ng/well Streptavidin-HRP (Life Technologies), followed by ELISA Femto Chemiluminescent Substrate (Thermo Fisher, Waltham, MA) and quantified on an Envision plate reader (PerkinElmer).

### Histology

Lymph node biopsies were fixed in 10% neutral buffered formalin for >24 hours, processed and embedded in paraffin, sectioned at 4 μm thick, and stained with hematoxylin and eosin. Sections were evaluated and tumors histologically classified according to World Health Organization classification criteria by a board-certified veterinary pathologist [30].

### Response assessment

Up to five target lesions were measured using calipers, weekly during the first four weeks on study, and every two weeks thereafter. Tumor response assessments were performed using the Response Evaluation Criteria for Peripheral Nodal Lymphoma in dogs (v1.0) [31]. A complete response (CR) was defined as disappearance of all disease on two measurements separated by a minimum period of 2 weeks. A partial response (PR) was defined as greater than 30% reduction in the sum of the longest diameters of target lesions documented by two assessments separated by at least 2 weeks. Progressive disease (PD) was defined as an increase of >20% in the sum of the longest diameters, using the smallest sum since initiation of therapy as a reference, or appearance of any new lesion(s). Stable disease (SD) was defined as the absence of criteria for either a response or progression. Objective response rate (ORR) was defined as the percentage of dogs with complete or partial responses. Progression free survival (PFS) was measured from day 0 until PD. Clinical benefit (CB) was defined as having SD, PR or CR for at least 28 days.

### Adverse events (AEs)

AEs were graded in accordance with the VCOG-CTCAE v1.1 criteria [27]; the AEs reported here are restricted to those considered as possibly, probably or definitely related to acalabrutinib administration. For the patients that died during the trial, a peer-reviewed pathologic exam was performed to investigate cause of death.

### Statistical analysis

For proliferation data, raw data were log transformed to reduce variance and skewness. Linear mixed effects models were applied to apoptosis data and the log-transformed proliferation data to account for the correlation of the observations from the same batch. A linear mixed effects model was used for analysis of the quantified western blots with dose dependent decrease (slope) in phosphorylation as the primary endpoint. Progression free survival (PFS) in dogs achieving PR versus SD was analyzed using log-rank test. P-value<0.05 was considered as significant. SAS 9.4 was used for those data analyses (SAS Institute Inc., NC).

## Results

### Activity of acalabrutinib against canine lymphoma cells in vitro

#### Acalabrutinib antagonizes BCR activity

**Fig 1:**
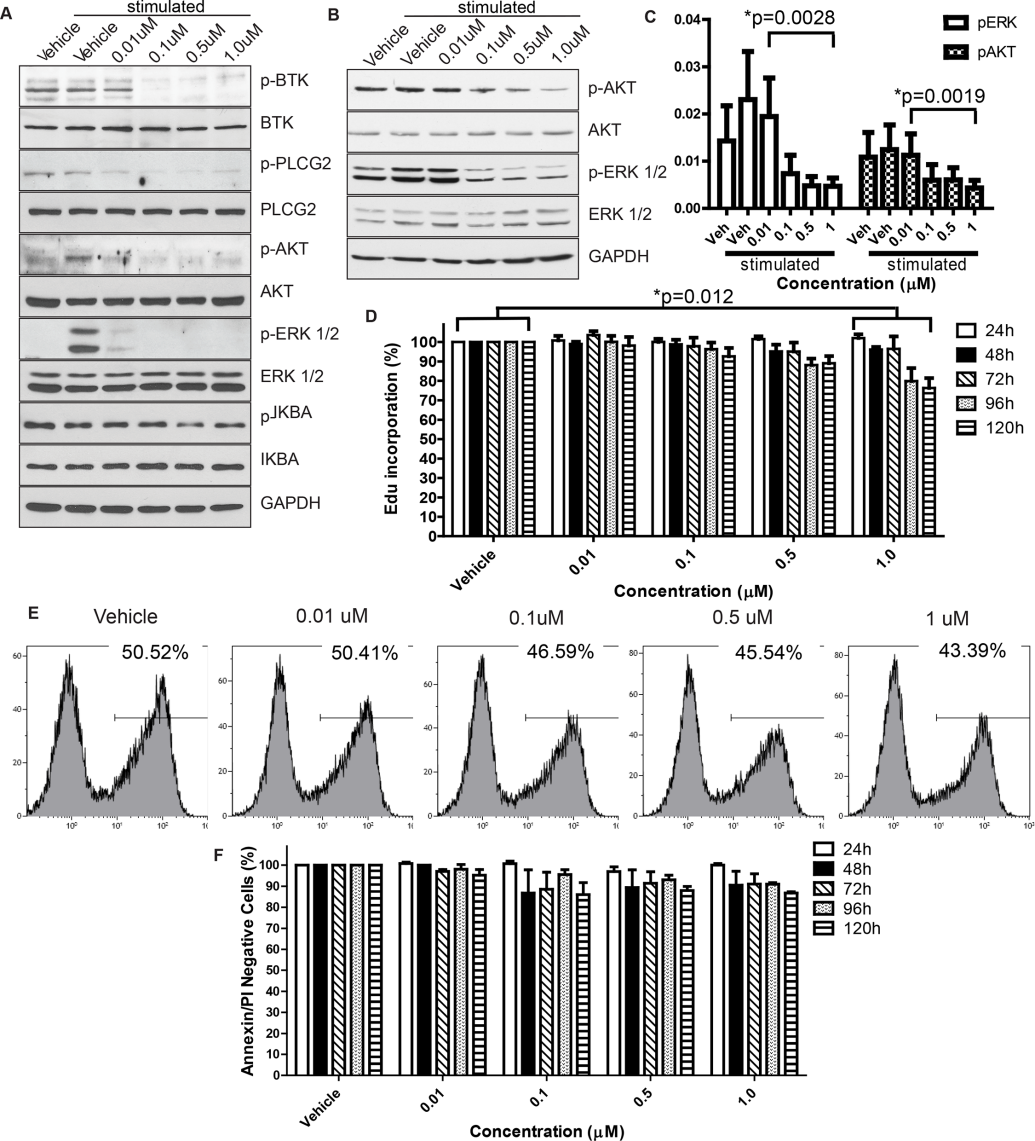
Effects of acalabrutinib inhibitors on canine lymphoma cells. Dose-dependent inhibition of BTK autophosphorylation, in addition to downstream targets, was observed via immunoblot at drug concentrations as low as 0.01μM following 1 hour of treatment with acalabrutinib in the canine B-cell lymphoma CLBL1 cell line (representative of 3 repetitions) (A) and primary canine lymphoma cells treated ex vivo (representative of 4 patients, separate from the clinical study population) (B). C. Densitometry quantification of the western blots from B. Bands of phospho-proteins are normalized to respective total proteins and loading control. There was a significant dose-dependent decrease in phosphorylation for p-ERK (P = 0.0028) and p-AKT (p = 0.0019). D. Dose-dependent reductions in cell proliferation following daily treatment with acalabrutinib in the canine CLBL1 B-cell lymphoma cell line. Results are the mean of 5 independent experiments. Raw data were log transformed to reduce variance and skewness. Linear mixed effects models were applied to apoptosis data and the log-transformed proliferation data to account for the correlation of the observations from the same batch. p = 0.012 E. Representative histograms showing a dose-dependent reduction in Edu incorporation from a single day at the 72 hour timepoint. F. Dose-dependent trend toward reductions in cell viability in CLBL1. Results are the mean of 3 independent experiments. Effects not statistically significant in a linear mixed effects model.

Herman and colleagues previously demonstrated inhibition of BTK and downstream targets in primary human CLL cells using ibrutinib [22]. We investigated whether similar pathway inhibition could be achieved using acalabrutinib in canine lymphoma cells. The canine B-cell lymphoma line CLBL1 was treated with varying concentrations of acalabrutinib for 1 hour, and then stimulated with anti-IgM and subjected to analysis of the BCR signaling pathway. Dose-dependent inhibition of BTK autophosphorylation and phosphorylation of downstream targets PLCG2, ERK, IKBA, AKT, and NFKB was observed at drug concentrations between 10 nM and 1 μM (Fig 1A). To confirm the results from CLBL1 cells, FNAs from 4 dogs with spontaneous B-cell lymphomas were tested. These dogs, which were not included in the clinical trial, were either treatment-naive or had received previous chemotherapy treatments. Treatment and analysis were performed as with CLBL1 cells, and similar antagonism of BCR signaling by acalabrutinib was observed regarding phosphorylation of BTK and downstream targets (Fig 1B and 1C).

#### Acalabrutinib inhibits cell proliferation and survival

In CLBL1 cells treated with acalabrutinib, there was significantly decreased cell proliferation compared to the vehicle control at concentration 1μM, and dose-dependent reductions in proliferation were observed for concentrations up to 1μM. In addition, the cells showed a significantly reduced trend of proliferation over the incubation time (Fig 1D and 1E). A reduced percentage of viable cells over the tested drug concentrations (negative for both PI and Annexin V, Fig 1F), was also observed following treatment with acalabrutinib. These effects, though modest, are comparable with those observed at physiologic concentrations in both primary CLL cells and DLBCL cell lines [32,33].

#### The gene expression pattern of CLBL1 resembles the ABC subtype of canine DLBCL

We next used GEP to sub-categorize the cell line CLBL1. Human DLBCL cases can be sub-categorized into activated B cell (ABC)-like or germinal-center B cell (GCB)-like subtypes on the basis of GEP [20]. In human DLBCL, ibrutinib treatment was more effective in patients with the ABC subtype (or non-GCB by immunohistochemistry), compared to GCB DLBCL patients [34]. Recently, canine B-cell lymphomas were classified into ABC and GCB subtypes, and prognostic differences (in PFS and OS) were similar to those observed with their human counterparts [20]. We performed GEP analysis on CLBL1 to characterize it relative to ABC or GCB subtypes within canine B-cell lymphoma. Microarray data from CLBL1 and [20] (published in GEO GSE43644) were analyzed side by side using the RMA method, and samples were clustered using centroid linkage. CLBL1 clustered with ABC subtype samples from GSE43664 (S1 Fig). Clustering was confirmed using Euclidian distance, Pearson correlation, and un-centered correlation. With each method, CLBL1 was more similar to the ABC than to the GCB subtype.

### Phase I study

#### Patient demographics

A dose-escalation study was initiated with acalabrutinib administered orally either once (QD) or twice daily (BID) at dosages between 2.5 mg/kg and 20 mg/kg in twenty companion dogs with spontaneously occurring B-cell lymphoma. Patient demographics are summarized in Table 1. Peripheral lymph node biopsy specimens were morphologically subclassified as DLBCL with either immunoblastic or centroblastic cytologic features (S2A and S2B Fig). One sample was classified as diffuse large cell morphology with nodular architecture (S2C Fig).

**Table 1:** Patient Demographics.

		*ACP-196*
		*(N = 20)*
**Age (Years)**	*Mean*	6.25
	*Median*	5.5
	*Range*	3–13
**Breed**	*Pure Breed*	14
	*Mixed Breed*	6
**Sex**	*Male Castrated*	7
	*Male Intact*	1
	*Female Spayed*	12
	*Female Intact*	0
**Weight (kg)**	*Mean*	24.74
	*Median*	20.7
	*Range*	7.7–51.8
**Prednisone**	*Yes*	16
	*No*	4
**Prior Chemotherapeutics**	*Yes*	14
	*No*	6
**Lymphoma Subtype**	*DLBCL—IB*	8
	*DLBCL–CB*	10
	*Not Available*	2

#### Pharmacokinetics and pharmacodynamics

**Fig 2:**
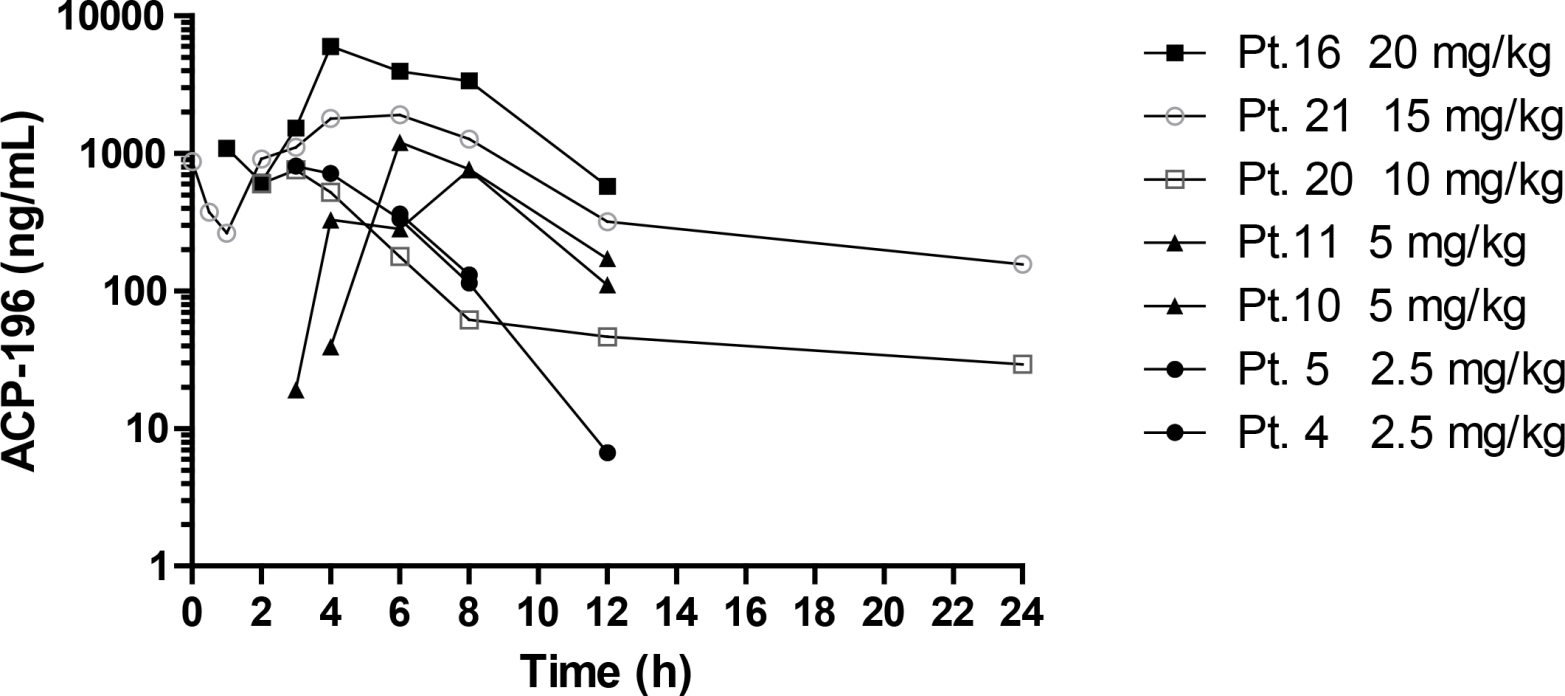
Pharmacokinetic data. Plasma levels of acalabrutinib were measured at 0.5, 1, 2, 4, 6, 8, 12 and 24 hours after oral dose administration from 7 patients on day 14. 1000 ng/mL = 2.1 μM.

Absorption was delayed in most patients with the first measureable levels of acalabrutinib detected between 3 and 6 hours post administration on day 14. Pharmacokinetic analysis was performed in a total of 7 dogs (2–3 dogs per cohort). The C_max_/Dose (mean±STD) was 195±94 ng/ml or 419±201 nM, and the median (range)T_max_ 6 (3,8) hours_._ Twelve hour exposures (AUC_0-12hr_) ranged from 3,086 to 31,445 hr*ng/ml with an average dose normalized AUC_0-12hr_ of 1,005±476 hr*ng/ml. Pharmacokinetic data are summarized in Fig 2.

BTK target occupancy was evaluated in peripheral blood and lymphoma cells from FNAs. Full BTK occupancy (>90% of available target) was achieved in peripheral B-cells at 3 hours after dosing on day 1 with a dosage of 2.5 mg/kg QD, for 5 of the 6 dogs in this cohort. A single dog with high peripheral B-cell count had 57% BTK occupancy on day 1, but had attained BTK occupancy of 94% prior to dosing on day 7. In samples taken at pre-dose on day 7 (*i*.*e*.,12 or 24 hours after prior dose administration), 83% to 99% BTK occupancy was observed among dogs in the 2.5 mg/kg QD cohort. A similar pattern was observed in higher dose cohorts, with complete BTK target coverage (Table 2).

**Table 2:** Percentage BTK target occupancy in peripheral blood B-cells and lymphoma aspirates of dogs following treatment with acalabrutinib. All values are presented as the average percentage BTK occupancy relative to control levels of BTK in matched pre-study samples. Due to poor sample quality the 5 mg/kg cohort was not included in the analysis. ND–not determined.

BTK Sample and Timepoint	Peripheral B cells		Fine Needle Aspirates	
	Day 1 3h	Day 7 predose	Day 1 3h	Day 7 predose
2.5 mg/kg QD	93	90	ND	ND
	(n = 6)	(n = 5)		
10 mg/kg BID	99	92	100	82
	(n = 2)	(n = 2)	(n = 2)	(n = 2)
15 mg/kg BID	97	96	95	84
	(n = 4)	(n = 2)	(n = 3)	(n = 1)
20 mg/kg QD	91	93	98	85
	(n = 3)	(n = 2)	(n = 3)	(n = 2)

#### Safety

Acalabrutinib was well tolerated by most patients, and maximum tolerated dose was not reached. Clinical observations related to disease, comorbid conditions, concomitant medications and research were not considered AEs. All clinical observations related to acalabrutinib were considered AEs and are listed in Table 3. Signs referable to the gastrointestinal system (anorexia, emesis, diarrhea and weight loss) and lethargy were the most commonly observed findings, regardless of attribution to research or to study drug. Gastrointestinal AEs were responsive to medical management or were self-limiting. One dog was de-escalated from 10 mg/kg BID to 10 mg/kg QD dosing due to gastrointestinal AEs.

**Table 3:** Adverse events listed by grade and frequency.

Dose Group	Adverse Events (number of events by grade)																											
	Anorexia				Diarrhea				Lethargy				Vomiting				Nausea				Seborrhea				Weight Loss			
	1	2	3	4	1	2	3	4	1	2	3	4	1	2	3	4	1	2	3	4	1	2	3	4	1	2	3	4
**1 (n = 6)**	2												1															
**2 (n = 5)**																												
**3 (n = 2)**	1								1				1								1							
**4 (n = 3)**			1										1															
**5 (n = 4)**	2		1		1								1				1								1			

Two dogs experienced severe adverse events during the study, both of which were considered unlikely to be related to acalabrutinib administration. Patient 17 was euthanized one week after initiation of treatment due to septic shock in the face of severe disease progression. The cause of sepsis was not identified. Patient 18 was hospitalized on day 3 for bacterial prostatitis and emphysematous cystitis, resulting in immediate discontinuation of dosing with acalabrutinib. This patient was euthanized on day 11. Necropsy of both patients revealed no evidence of toxicity from acalabrutinib.

#### Response to therapy

**Fig 3:**
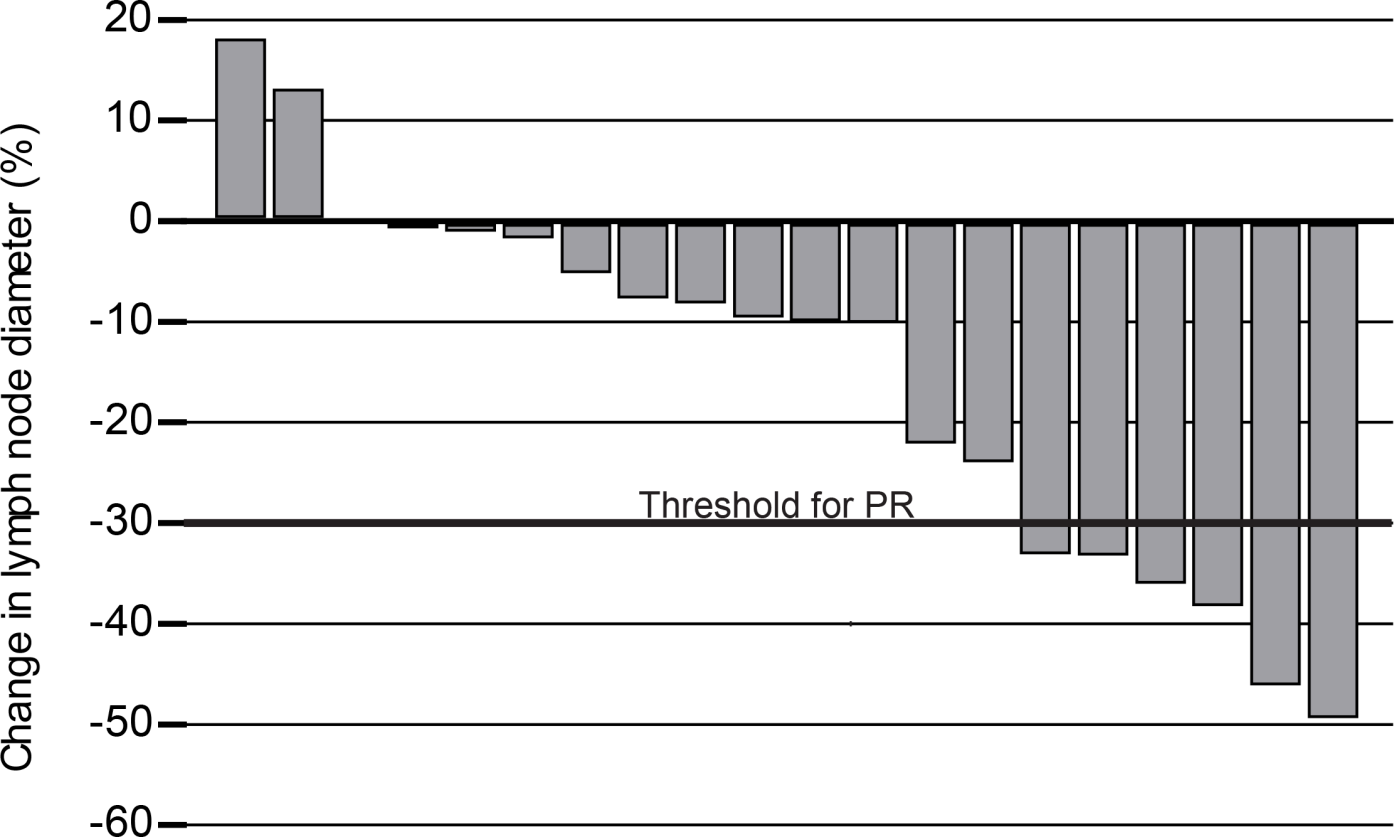
Reduced target lesion size in acalabrutinib treated dogs. Waterfall plot showing percentage decrease in mean sum of longest diameter of index lymph nodes as compared to baseline measurements.

**Fig 4:**
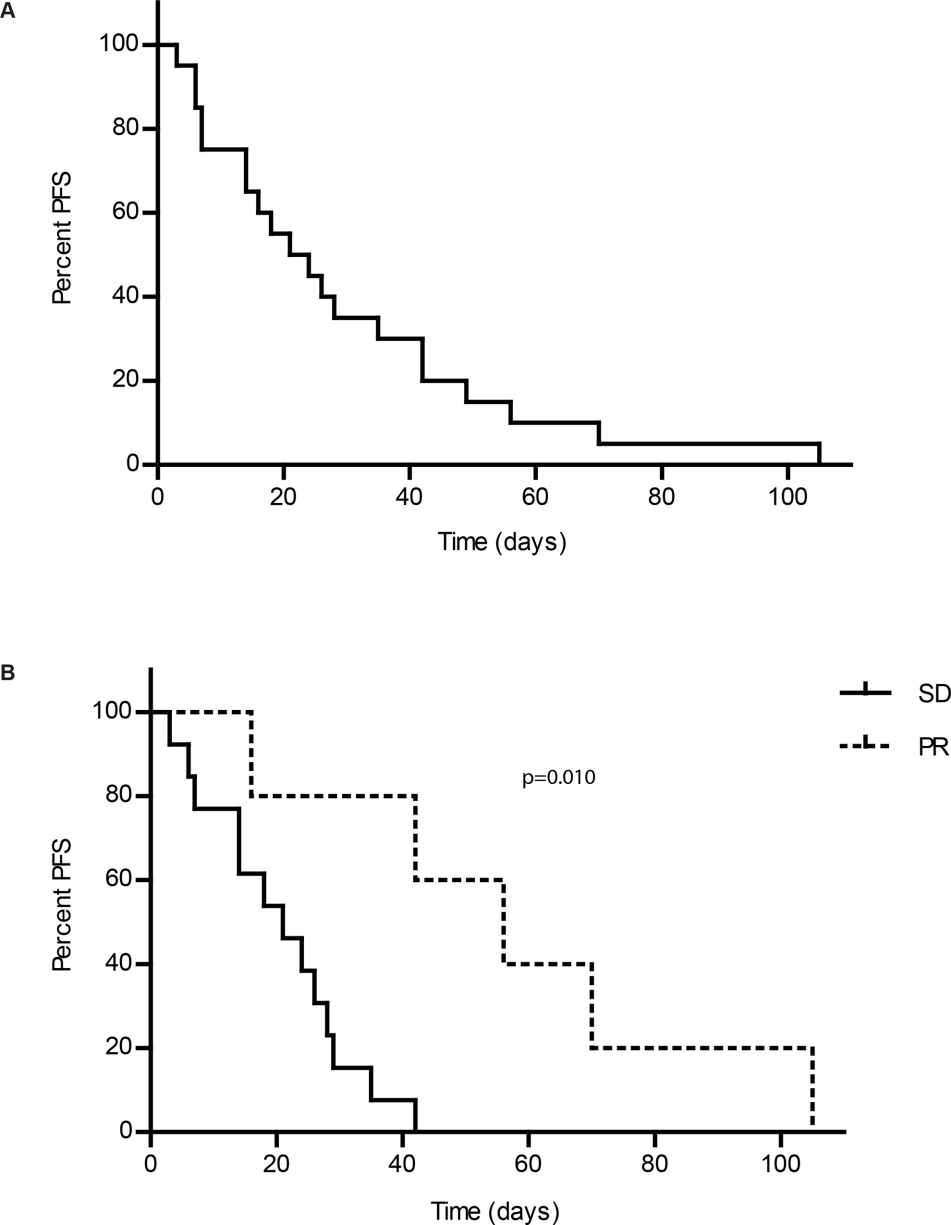
**PFS.** Kaplan-Meier curves showing overall PFS (A) and PFS of dogs achieving SD compared with PR (B). Dogs achieving PR survived significantly longer than those achieving SD (p = 0.010).

Most dogs experienced a reduction in target lesion (lymph node) size from baseline, as shown in Fig 3. Responses are summarized in Table 4A. Clinical benefit (PR or SD ≥28 days) was observed in 30% of patients. Median PFS for all patients was 22.5 days (Fig 4A). There was little variability in PFS when patients were stratified on relapse status at time of enrollment (Table 5). However, dogs achieving PR had significantly longer PFS (56 days) than those with SD (22.5 days) (Table 6, Fig 4B). In addition, some dogs with disease progression were escalated to higher doses of acalabrutinib either QD or BID. Notably, dose escalation resulted in renewed or sustained clinical benefit in five of six dogs (S1 Table).

**Table 4:** Clinical Response Rates.

Dose Group	Study ID	Histologic Subtype	Best Response	Objective Response	PFS
2.5 mg/kg QD	1	DLBCL	-0.55%	SD	14
	2	DLBCL-CB	-1.60%	SD	24
	3^#^^P^	DLCBL-CB	-38.01%	PR	105
	4^P^	DLCBL-IB	-8.06%	SD	26
	5^#^^P^	DLCBL-CB	-35.90%	PR	42
	6^P^	DLCBL-CB	-0.88%	SD	28
5 mg/kg QD	7^#^^p^	DLBCL-CB	-7.54%	SD	42
	8^p^	DLBCL-IB	-5.05%	SD	35
	9^P^	DLBCL	-9.85%	SD	21
	10^#^^P^	DLBCL-IB	-23.81%	SD	49
	11^#^^p^	DLCBL-CB	-9.42%	SD	7
10 mg/kg BID	12^#^^P^	DLCBL-CB	-49.23%	PR	56
	20^p^	DLCBL-CB	-7%	SD	18
20 mg/kg QD	14	DLCBL-CB	-22%	SD	14
	15^P^	DLBCL-IB	13%	PD	6
	16^p^	DLBCL-IB	-33%	PR	16
15 mg/kg BID	17^+^^P^	DLBCL-IB	-33.10%	PD	7
	18^P^	DLCBL-CB	-10%	SD	11
	21^p^	DLBCL-IB	-46%	PR	70
	24	DLBCL-IB	17.72%	PD	7

Patient response separated by treatment cohort is shown. Best response to treatment indicates the greatest percent reduction in mean sum of target lesions.

^#^This response was attained after dose escalation for some dogs. See S1 Table for details.

^P^ These patients were administered prednisone prior to enrollment and during the trial.

^p^ These patients began prednisone during the trial.

^+^This patient is classified as PD due to progression in non-target organs identified at necropsy.

**Table 5:** Median PFS and OR of Treatment Naïve and relapsed Patients.

	Median PFS	Objective Response
All patients	22.5	5 PR, 12 SD, 3 PD
Relapsed	19.5	4 PR, 7 SD,1 PD
Treatment Naïve	25	1 PR, 6 SD, 1 PD

PFS is measured in days.

**Table 6:** Median PFS and OR of Patients Achieving PR and SD.

Patient Response	Median PFS
PR	56*
SD	22.5*

PFS is measured in days

*p = 0.010

## Discussion

BTK is a vital component of BCR signaling, and targeting BTK is clinically efficacious in several hematologic malignancies, including B-cell lymphomas [34–37]. Although treatment with ibrutinib has demonstrated durable responses in some B-cell malignancies, notably CLL, ibrutinib has off-target activities that may limit the potential uses of this therapeutic. For example, ibrutinib irreversibly inhibits interleukin-2 inducible T-cell kinase [38], which mediates signaling essential for NK-cell antibody dependent cellular cytotoxicity (ADCC)[39]. It has been reported that concurrent administration of ibrutinib antagonizes rituximab-mediated ADCC in vitro and in a murine lymphoma model [40], and thus, combination of ibrutinib with monoclonal antibodies may not result in optimal efficacy. Additionally, the epidermal growth factor family receptors can be inhibited by ibrutinib [41]. Some common adverse events observed with ibrutinib treatment include skin rash and gastrointestinal effects, including diarrhea, vomiting, and anorexia. Similar toxicities are observed with epidermal growth factor receptor inhibitors [42,43]. Therefore, BTK inhibitors with greater target specificity could prove beneficial for patients if they result in fewer off-target effects.

As large animal models of lymphoma are scarce, we elected a canine model to evaluate safety and efficacy of acalabrutinib. Spontaneous cancers in dogs recapitulate their human counterparts with respect to biology, clinical behavior, molecular profiles, and conserved cytogenetic abnormalities, in addition to response and resistance to therapy [44]. As tumors develop naturally in animals with competent immune systems, the effects of novel agents on tumor growth and metastasis can be modeled more accurately, compared with mouse xenograft studies. Additionally, in spontaneously occurring cancer in dogs, the tolerability and efficacy of novel agents can be evaluated in outbred patients of various ages and co-morbidities. There are multiple examples where leveraging the dog model in preclinical drug development has informed the development path by providing pharmacokinetic/pharmacodynamic information, establishing dosing regimens, validating targets and identifying adverse events, and ultimately informing subsequent clinical studies in people [17,45,46].

Additional support for canine B-cell lymphoma as a model of human DLBCL comes from its classification into ABC and GCB subtypes [20] and the clustering of GEP from the canine CLBL1 cell line with ABC subtype of DLBCL. These fundamental similarities between canine and human lymphoma underscore the utility of comparative and translational research, providing the opportunity to target biochemical pathways critical to lymphoma pathogenesis and allowing preclinical assessment of novel therapeutics in a spontaneously occurring disease model.

Our *in vitro* results show that BCR signaling activates BTK and downstream targets in canine lymphoma cells, and inhibition of this signaling decreases proliferation and survival of CLBL1 cells. This response to BTK inhibition is similar to that demonstrated with ibrutinib in many types of human NHL [22,47–49]. Other mechanisms of action by acalabrutinib, such as autophagy, were not evaluated but could play a role in the drug’s efficacy and should be explored in future studies.

We evaluated the safety and efficacy of acalabrutinib in companion dogs of various genetic backgrounds with spontaneous B-cell lymphoma. Acalabrutinib pharmacokinetics following oral dosing in dogs were sufficient to achieve plasma concentrations expected to elicit the desired anti-BTK pharmacodynamic effects. Oral doses up to 15 mg/kg BID were well-tolerated, and biologic activity was observed in all cohorts. Full BTK occupancy was observed 3 hours after dosing in peripheral blood B-cells and FNA lymphoma samples obtained from all cohorts. These results demonstrated rapid tumor penetration by acalabrutinib. Trough (pre-dose) BTK occupancy evaluated in peripheral blood B-cells and lymphoma FNAs on study day 7 remained high, reflecting steady state inhibition of the pathway. The ability to obtain matching tissue and blood samples at multiple timepoints is one advantage of using this dog model.

Clinical benefit was observed in 30% of patients. Notably, dogs with either treatment-naïve or drug-resistant NHL experienced clinical benefit. The administration of prednisone to 16 patients represents one potentially confounding variable to the clinical benefit and response data. However, 10 of 16 dogs had experienced disease progression in the face of prednisone administration prior to study enrollment, detracting from the potential anti-neoplastic benefits these patients may have received from prednisone. Interestingly, this subset of 10 patients accounts for three of the most pronounced and durable responses. It is also interesting that in nearly all dogs that had dose escalations, additional clinical benefit was observed. This finding suggests clinical benefit may be dose-dependent, and because acalabrutinib was well-tolerated, higher doses may be better suited to use in follow up studies.

Results of the current study are comparable with early experiments with ibrutinib, in which a small cohort of dogs with spontaneous B-cell lymphoma was treated with dosages of 2.5 to 20 mg/kg QD. Twenty percent(1/5) of the dogs with DLBCL achieved a PR in the ibrutinib studies [17], compared with 25% in the present study. ORR in the present study is also comparable with that observed in ibrutinib-treated DLBCL patients [50] (both are 25%), a finding which lends additional support to the translational relevance of this model.

PFS appears abbreviated in this study (22.5 days). Unlike CLL, canine lymphoma is a rapidly progressive malignancy with median OS of only 4–6 weeks in newly-diagnosed patients without treatment. It is also important to note that two-thirds of dogs were chemotherapy refractory, and survival in these relapsed patients is often as short as 28 days, even with aggressive cytotoxic regimens (reviewed in [51]). Furthermore, early stage (stage 1) patients were excluded from the study. Taking this into consideration, ORR and PFS are more favorable than they appear at first glance. Additionally, the data suggest that this therapy may work best in a subset of dogs (the 5 dogs achieving PR). Preliminary studies in human DLBCL suggest factors such as molecular subtype (ABC vs. GCB) as well as specific mutations (MYD88, CD79, CARD11 and TNFAIP3) may predict response to ibrutinib [10,11]. We are currently pursuing additional studies to determine whether such molecular differences exist between our canine patients achieving a PR and those with SD or PD.

The toxicity profile of acalabrutinib is favorable in comparison to standard chemotherapy regimens often used for canine lymphoma. The most common AEs were mild and gastrointestinal in nature (anorexia, vomiting) and responded to medical interventions or temporary drug discontinuation. Therapy with cyclophosphamide, doxorubicin, vincristine, and prednisone combinations yields frequent and occasionally severe toxicities in dogs. Whereas this study focused on acalabrutinib monotherapy, the favorable AE profile of acalabrutinib suggests combination strategies may support reduced doses of standard chemotherapeutic agents. Combination with other targeted therapies could yield an increase in response rates or durations. Such therapeutic strategies may help alleviate AEs associated with chemotherapy, in addition to improving responses and survival.

This work demonstrates histologic, biologic and molecular similarities between canine and human NHL, including classification of the ABC molecular subtype of CLBL1 cells. It also reproduces the expected biologic responses to BTK inhibition in vitro and in vivo with canine B-cell lymphoma. The value of spontaneously occurring canine lymphoma as a model for human DLBCL is demonstrated. Using this model, acalabrutinib was orally administered at doses and schedules that provided important information about biologic action, tolerability, and anti-tumor efficacy in an aggressive disease setting. Based on these promising monotherapy results, further evaluation of acalabrutinib in combination with other agents in this spontaneous B-cell lymphoma model is merited.

## Supporting Information

S1 Fig CLBL1 clusters with ABC-like canine lymphoma subtype. Two-way hierarchical clustering that combines CLBL1 with canine samples from GSE43664. In the colorbar at the top, CLBL1 is pink, and the samples from GSE43664 are blue for group 1 (GCB-like) and brown for group 2 (ABC-like).
(EPS)

S2 Fig Histopathology of peripheral lymph node biopsies from dogs enrolled in the Acalabrutinib clinical trial. All patients were morphologically classified as DLBCL with either centroblastic (A) or immunoblastic (B) morphology. A single patient was noted to have marked nodular architecture reminiscent of follicular structures (C). (Hematoxylin and eosin).
(EPS)

S1 Table Dose Escalation. Dose escalation resulted in prolonged CB for 5 of 6 patients. (Patient 11 did not attain CB because the duration of SD was only 11 days.)
(XLSX)
